# ChatGPT as a Clinical Decision Maker for Urolithiasis: Compliance with the Current European Association of Urology Guidelines

**DOI:** 10.1016/j.euros.2024.08.015

**Published:** 2024-09-16

**Authors:** Ali Talyshinskii, Patrick Juliebø-Jones, B.M. Zeeshan Hameed, Nithesh Naik, Kinju Adhikari, Ulanbek Zhanbyrbekuly, Lazaros Tzelves, Bhaskar Kumar Somani

**Affiliations:** a Department of Urology, Astana Medical University, Astana, Kazakhstan; b Department of Clinical Medicine, University of Bergen, Bergen, Norway; c Department of Urology, Father Muller Medical College, Mangalore, Karnataka, India; d Department of Mechanical and Industrial Engineering, Manipal Institute of Technology, Manipal Academy of Higher Education, Manipal, Karnataka, India; e Department of Urology, HCG Cancer Centre of Bangalore, Bangalore, Karnataka, India; f Department of Urology, University of Athens, Athens, Greece; g Department of Urology, University Hospital Southampton NHS Trust, Southampton, UK; h Department of Urology, Haukeland University Hospital, Bergen, Norway; i EAU Young Academic Urology Urolithiasis Group, Arnhem, Netherlands

**Keywords:** Generative pretrained transformer, Urolithiasis, Diagnosis, Treatment, Clinical decision

## Abstract

**Background and objective:**

Generative artificial intelligence models are among the most promising and widely used tools used in health care. This review investigates GPT-4 answers to decision-making questions regarding the diagnosis and treatment of urolithiasis across several clinical settings and their correspondence to the current European Association of Urology (EAU) guidelines.

**Methods:**

In March 2024, the GPT-4 model was asked 11 questions, containing a brief description of a patient with urolithiasis. All questions were grouped according to urolithiasis care step: diagnosis, urgent care, scheduled intervention, and metaphylaxis. When responses were received, compliance with the current EAU guidelines was assessed by experienced urologists.

**Key findings and limitations:**

Although all responses were provided with information that corresponded to EAU guidelines, six of the 11 answers were associated with missed guideline–provided parts, and incorrect data were given in eight of the 11 answers. GPT-4 is relatively safe in the initial diagnostic flow of patients suspected of having stones within the urinary tract and during treatment planning; however, its understanding of all the nuances of metaphylaxis leaves much to be desired and is far from the dogma given in the EAU guidelines. Moreover, GPT-4 knowledge of strategy and algorithm is not always aligned with the EAU guidelines.

**Conclusions and clinical implications:**

Despite the fact that from the perspective of patients with urolithiasis, GPT-4 is capable of answering their questions well, the specificity of questions from urologists is labor intensive for its current version, and necessitates the ability to interpret it correctly and further attempts to improve it. While some aspects of diagnostics are more accurate, these struggle with surgical planning and algorithms in line with the EAU guidelines.

**Patient summary:**

The generative artificial intelligence (AI) model GPT-4 is capable of answering urology-related questions, but lacks detailed responses. Although some aspects of the diagnostics are accurate, knowledge of surgical planning is not in line with the European Association of Urology guidelines. Future improvements should focus on efforts to enhance the accuracy, reliability, and clinical relevance of AI tools in urology.

## Take Home Message

GPT-4 is able to answer patient questions adequately but lacks detailed responses. It does not cover the depth of advice covered in the European Association of Urology guidelines. Future versions should consider using guidelines in the training data.

## 1 Introduction

The modern era is characterized by the seamless integration of digital technologies into all facets of health care. Artificial intelligence (AI) is among the most promising and widely used tools in this field. AI allows for the optimization of patient counseling and diagnosis of various diseases, as well as the prediction of events following therapy [1]. The real frostings on the AI cake are generative AI models, which allow us to receive current information in both text and graphic forms based on the user's request. The most well-known of these models is OpenAI's generative pretrained transformer (GPT) model. The makers introduced ChatGPT in November 2022, utilizing GPT-3.5, which was trained on 175 billion parameter tests. GPT-4 was launched with a model size of 170 trillion parameters and support for the picture inputs. The GPT-4 design uses a neural network to parse natural language and generate replies based on the input context.

The advantage of GPT-4 over its GPT-based predecessors stems from its capacity to generate refined and extremely intelligent responses in several languages using advanced modeling. This technology is used widely in the field of urology. Thus, GPT-4 offers numerous opportunities for urologists, ranging from streamlining doctors' processes to improving patient contact and providing decision support tools [2]. This system, which functions primarily as a knowledge database, is extremely good at answering inquiries on pediatric urology, benign prostate hyperplasia, urolithiasis, prostate cancer, and andrology [3], [4], [5]. A recent study found that ChatGPT (August 3 version) accurately and properly answered >95% of urolithiasis-related inquiries [6]. Kim et al [7], on the contrary, noticed a negative change in perception among urolithiasis patients following the receipt of the AI chatbot program's explanatory note and concluded that the AI chatbot program's explanatory note could cause an unpleasant change in AI perception. This disparity was observed only during the process of alerting patients. Given that the diagnosis and treatment of urolithiasis is a constantly evolving and multifaceted area of urology, it is critical to assess the role of the GPT in the correct interpretation of patient data and clinical decision-making to provide an objective assessment of this technology and understand its maturity for use not only by patients, but also by physicians. In this regard, this study investigated GPT-4 answers to decision-making questions in the diagnosis and treatment of urolithiasis across several clinical settings and its correspondence to the current European Association of Urology (EAU) guidelines.

## 2 Methods

One of the authors (A.T.) conducted a poll on the developer’s official website (https://chat.openai.com/) in March 2024 for the GPT-4 model. The latter was asked 11 similar questions, containing a brief description of a patient with urolithiasis. Notably, all requests were imagined and approved by a senior urologist (B.K.S.). All questions are grouped according to the steps of urolithiasis care: diagnosis (numbers 1 and 2), urgent care (numbers 3–5), scheduled intervention (numbers 6–9), and metaphylaxis (numbers 10 and 11; Table 1).

**Table 1:** Description of questions

Question number	Aspect	Question content
1	Diagnosis	A 35-yr-old female presents with severe flank pain and hematuria. Which imaging modality should be used first? Which laboratory should be initiated?
2	Diagnosis	A 49-yr-old woman performed a routine ultrasound of her kidneys and accidentally discovered a stone measuring 14 mm in the left kidney pelvis. There are no complaints. From the anamnesis it is known that she drinks little liquid per day and likes salty foods. Which urine and blood laboratory is necessary to evaluate this patient?
3	Urgent care	A 55-yr-old female with a history of recurrent urinary tract infections presents with flank pain and fever. CT shows a 6 cm stone in the solitary kidney. What is the treatment plan?
4	Urgent care	A 45-yr-old male presents with recurrent episodes of renal colic. CT revealed a stone of 7 mm in the ureterovesical junction. Patient suffers from vomiting, fever. Last episode was 1 yr ago, and stone was given for analysis and composed of 100% uric acid. How should we start to treat this urgent condition and what to do next?
5	Urgent care	A 43-yr-old man suffers from new-onset renal colic and was taken to the hospital, where he underwent noncontrast abdomen CT. A stone was identified in the upper third of the ureter with dimensions of up to 9 mm and a density of 1100 HU. He denies chills, and there are no signs of systemic inflammation according to laboratory data. What is the treatment strategy for this patient?
6	Scheduled intervention	A 51-yr-old man consulted a urologist as planned with flank pain on the right. From the anamnesis, it is known that he suffers from frequent recurrence of stone formation but does not follow the recommendations of doctors, and the composition of past stones is unknown. He also suffers from coagulopathy and hepatomegaly. A CT scan was performed—several stones of the right kidney were detected in the pelvis (2.3 cm) and in the calyces of the upper and middle groups (up to 1.4 cm) with a density of up to 700 HU. What are the tactics for further examination? Is surgical treatment indicated? If yes, what is the procedure of choice?
7	Scheduled intervention	A 42-yr-old man complaining of aching pain in the left lower back consulted a urologist. An ultrasound was performed, and a stone of the lower calyceal group up to 10 mm was detected. According to CT results, the stone density is 900 HU; the length of the lower infundibulum is <10 mm, and the distance from the skin to the stone is 9 cm. According to laboratory data, there are no signs of UTI or coagulopathy. The patient's body mass index is within normal values (21). What is the treatment strategy for this patient?
8	Scheduled intervention	A 36-yr-old woman underwent abdomen noncontrast CT, and a stone of the lower group of calyces of the left kidney measuring 19 mm and density 1300 HU was detected accidentally. The patient's body mass index is >30. What additional examination is necessary and what are the surgical options?
9	Scheduled intervention	A 55-yr-old man consulted a urologist as planned with flank pain on the right. From the anamnesis, it is known that he suffers from frequent recurrence of stone formation but does not follow the recommendations of doctors, and the composition of past stones is unknown. He also suffers from coagulopathy and hepatomegaly. A CT scan was performed and revealed a solitary stone of the right kidney in the pelvis up to 1.7 cm, with a density of up to 600 HU. Is surgical treatment indicated? If yes, what is the procedure?
10	Metaphylaxis	A 33-yr-old woman consulted a urologist with spontaneous stone passage. This is the first time such an episode has happened. The patient states that she suffers from intermittent pain in the lumbar region on both sides. She was sent for an ultrasound of the kidneys—a horseshoe-shaped kidney was detected. Ultrasound determines increased papillary density within PCS. Which risk group for recurrent urolithiasis does the patient belong to? Is it necessary to submit a stone to determine its mineral composition? Is it necessary to perform a 24-h urine analysis? What parameters need to be determined and what are the reference values?
11	Metaphylaxis	A 33-yr-old woman consulted a urologist with spontaneous stone passage. This is the first time such an episode has happened. The patient claims that she has never suffered from flank pain, there has been no change in the color of urine, and none among her relatives suffers from KSD. She was sent for an ultrasound of the kidneys—no developmental anomalies and no signs of the presence of stones were detected. Which risk group for recurrent urolithiasis does the patient belong to? Is it necessary to submit a stone to determine its mineral composition? Is it necessary to perform a 24-h urine analysis? What parameters need to be determined and what are the reference values?

CT = computed tomography; KSD = kidney stone disease; UTI = urinary tract infection.

To imitate a scenario in which GPT-4 is utilized by an average urologist with no prior experience, the inquiry included only the following queries and no additional follow-up questions to assess both GPT-4 models' primary ability to analyze raw data. When responses were received, their compliance with the current EAU guidelines was assessed: the presence of up-to-date information, absence of up-to-date information, and presence of incorrect information were determined independently by two experienced urologists (B.K.S. and B.M.Z.H.).

## 3 Results

Queries and responses provided by GPT-4 are listed in Table 2.

**Table 2:** Answers and their compliance with guidelines

Case number	GPT-4			
	Response	Presence of correct information	Some correct information missing	Presence of incorrect information
1	The first-line imaging modality for suspected urolithiasis is a noncontrast helical (spiral) CT scan of the abdomen and pelvis. This type of a CT scan is highly sensitive and specific for detecting kidney stones, can identify the size and location of the stones, and can help guide management decisions. It has the advantage of being rapid and does not require the administration of intravenous contrast, which is beneficial in patients with potential renal impairment. An alternative, particularly in pregnant women due to concerns about radiation exposure, is an ultrasound of the kidneys and bladder. Ultrasound can identify hydronephrosis (swelling of a kidney due to a build-up of urine) and large stones but may miss smaller stones. It is safe in pregnancy and can be a good initial choice in this specific population. Several laboratory tests should be initiated to assess the patient's renal function and risk of infection, and to guide further management: 1. Complete blood count: To check for signs of infection or significant blood loss 2. Basic metabolic panel or comprehensive metabolic panel: To evaluate kidney function (creatinine, BUN) and electrolytes 3. Urinalysis: Crucial for detecting hematuria, crystalluria (presence of crystals in urine, which might indicate the type of stone), or signs of infection (leukocyte esterase, nitrites) 4. Urine culture: If there are signs of infection on urinalysis, to identify the causative organism and guide antibiotic therapy 5. Blood cultures: If the patient shows signs of sepsis or severe infection 6. Serum calcium, uric acid, and phosphorus levels: these can help determine the type of stone and underlying metabolic disorders Special considerations: 1. In pregnant women, an ultrasound is preferred due to concerns about radiation exposure from CT scans. 2. In children, both ultrasound and low-dose noncontrast CT scans are considered to minimize radiation exposure. 3. Patients with impaired renal function or allergies to contrast materials should avoid contrast-enhanced imaging studies.	+	–	+
2	*Urine tests* 1. Urinalysis: To check for hematuria (blood in the urine), crystalluria (presence of crystals, which might indicate the type of stone), pH (which can suggest the type of stone), and signs of infection (eg, leukocyte esterase, nitrites) 2. 24-h urine collection: This test evaluates the volume of urine produced in a day and measures the levels of various substances that can form stones if present in excess amounts. The key components include the following: (a) Calcium: High levels can indicate a risk for calcium stones. (b) Uric acid: Elevated uric acid can lead to uric acid stones. (c) Oxalate: High oxalate levels are associated with oxalate stones. (d) Citrate: Low levels can promote stone formation. (e) Sodium: Reflects salt intake, which can influence calcium and other mineral levels in the urine. (f) pH: Abnormal urine pH can suggest certain types of stones (eg, acidic urine with uric acid stones, alkaline urine with struvite stones). *Blood tests* 1. Basic metabolic panel or comprehensive metabolic panel: To evaluate kidney function (creatinine, BUN) and electrolytes. Abnormalities can indicate underlying metabolic conditions contributing to stone formation. 2. Serum calcium: High levels can be associated with hyperparathyroidism, leading to increased calcium stone risk. 3. Serum uric acid: To identify hyperuricemia, which can contribute to uric acid stones 4. Phosphorus: Abnormal levels can be involved in stone formation. 5. Parathyroid hormone (PTH): If serum calcium is elevated, measuring PTH can help diagnose hyperparathyroidism, a potential cause of calcium stones. These tests help identify metabolic abnormalities that could contribute to stone formation and guide dietary or pharmacologic interventions to prevent future stones. Given the size of the stone (14 mm), it is unlikely to pass spontaneously, and she may require intervention. However, as she is asymptomatic currently, a conservative approach with monitoring may initially be considered while addressing modifiable risk factors such as increasing fluid intake and reducing dietary sodium. The decision regarding intervention would also take into account other factors such as stone composition (if known), location, and any signs of obstruction or kidney function impairment.	+	+	–
3	*Immediate management* 1. Stabilization: Initial management should focus on stabilizing the patient. This includes intravenous (IV) fluids to maintain hydration and correct any electrolyte imbalances, and IV antibiotics to cover for likely pathogens causing the UTI and potential sepsis. Blood cultures should be taken before initiating antibiotics. 2. Pain management: IV analgesics may be needed to manage flank pain. *Urgent intervention* 1. Relieving obstruction: With a large stone (6 cm) causing obstruction in a solitary kidney, urgent decompression is necessary to preserve renal function and control infection. Options include the following: (a) PCN: A procedure where a catheter is placed directly into the kidney through the skin to drain urine externally. This can quickly relieve obstruction. (b) Ureteral stenting: Insertion of a stent via cystoscopy from the bladder into the ureter to bypass the obstruction. This allows urine to flow from the kidney to the bladder. The choice between PCN and stenting depends on various factors including the patient's clinical status, local expertise, and specifics of the anatomy or infection. In cases of severe infection or where immediate relief is critical, PCN might be preferred due to its ability to decompress the kidney rapidly and facilitate drainage of infected material. *Definitive treatment* After stabilization and initial management of infection and obstruction: 1. Stone removal: A 6-cm stone in a solitary kidney will require definitive treatment for removal once the patient is stabilized and infection is controlled. Options include the following: (a) PCNL: This is a minimally invasive surgery performed through a small incision in the back to remove large kidney stones directly from the kidney. Given the size of the stone, PCNL is often the treatment of choice. (b) Flexible URS and laser lithotripsy: Though used less commonly for stones of this size due to limitations in how much can be treated safely in one session, it may be considered based on stone composition, location, and patient factors.	+	+	–
4	*Immediate management* 1. Pain relief: Renal colic is extremely painful, so initial treatment should focus on pain management. NSAIDs are typically the first-line treatment for pain relief in renal colic, but opioids may be necessary for adequate analgesia if NSAIDs are contraindicated or insufficient. 2. Hydration: IV fluids can help maintain hydration, especially if the patient is vomiting. However, aggressive hydration is not recommended as it may increase renal pelvis pressure and pain without improving stone passage rates. 3. Infection management: Fever suggests the possibility of an infection, which can be a life-threatening condition (urosepsis) in the presence of an obstructing stone. Immediate empirical broad-spectrum antibiotics should be initiated after obtaining blood and urine cultures. The choice of antibiotics should be guided by local resistance patterns and adjusted based on culture results. 4. Imaging: Noncontrast helical CT scan is the gold standard for diagnosing urinary stones and assessing their size, location, and possibly composition. However, since this has already been done, further imaging may be necessary only if complications or alternative diagnoses are suspected. *Urgent intervention* Given the size of the stone (7 mm), its location, and the presence of systemic symptoms (fever), this situation warrants urgent intervention to relieve obstruction, especially in the context of possible infection: 1. Ureteral stenting or PCN: Either of these procedures can be used to relieve obstruction, allowing urine to bypass the stone. They also help mitigate the risk of sepsis while definitive stone management is planned. *Definitive stone management* After stabilization and resolution of any infection: 1. Stone removal: Options include the following: (a) URS with laser lithotripsy: A small scope is passed through the urethra and bladder into the ureter where the stone can be visualized and fragmented using a laser. The fragments can then be extracted or passed naturally. (b) ESWL: This noninvasive option uses shock waves to break the stone into smaller pieces that can be passed in the urine. However, its effectiveness can be limited for stones at the ureterovesical junction or in obese patients. Given that the stone is composed of uric acid, medical dissolution therapy could also be considered if there were no signs of infection or obstruction. This involves alkalinization of the urine with oral potassium citrate to dissolve uric acid stones. However, with current symptoms indicating potential infection and obstruction, immediate surgical intervention is preferred. *Long-term management* Preventive strategies are crucial to avoid recurrence: 1. Dietary and lifestyle modifications: Increase fluid intake to produce at least 2.5 l of urine daily, reduce intake of purine-rich foods (eg, red meat, seafood), and adopt a diet that promotes alkaline urine. 2. Medication: Allopurinol may be prescribed to reduce uric acid levels if there is evidence of hyperuricosuria. 3. Follow-up: Regular follow-up with imaging to monitor for new stone formation, metabolic evaluation to identify risk factors for stone formation, and urine pH monitoring to ensure effectiveness of alkalinization therapy.	+	+	–
5	*Immediate symptomatic management* 1. Pain relief: The primary immediate concern is to manage the renal colic pain. NSAIDs are the first-line treatment due to their dual action of pain relief and reduction of ureteral edema, which can facilitate stone passage. If NSAIDs are contraindicated or insufficient, opioids may be used as a second-line option. 2. Hydration: Adequate hydration is encouraged unless contraindicated, to help facilitate stone passage. However, excessive hydration should be avoided as it does not improve stone passage rates and may increase discomfort. 3. MET: Alpha-blockers, such as tamsulosin, can be used to relax the smooth muscle in the ureter, potentially facilitating the passage of the stone. While the effectiveness of MET has been debated, it is often considered for stones <10 mm that are located in the ureter. *Definitive stone management* Given the size (9 mm) and location (upper ureter) of the stone, along with its high density (1100 HU), spontaneous passage is less likely. Stones larger than 6 mm and those located in the upper ureter have a lower rate of spontaneous passage. Additionally, the high density of the stone suggests that it may be more resistant to fragmentation by ESWL. Therefore, the treatment options include the following: 1. Observation with MET: In selected patients who prefer to avoid intervention and have manageable symptoms, observation with MET can be an option for a limited period, typically up to 4–6 wk. During this time, if the stone passes or symptoms remain controlled, further intervention might not be necessary. However, this approach requires patient compliance with follow-up and understanding of potential complications. 2. URS with laser lithotripsy: This is a common and effective treatment for ureteral stones that are unlikely to pass spontaneously. A small scope is passed through the urethra into the ureter, and the stone is fragmented using a laser. The fragments can then be removed or left to pass naturally. URS is particularly effective for stones with high density, as seen in this case. 3. ESWL: While ESWL is a noninvasive option that uses shock waves to break up stones, its effectiveness decreases as the stone size increases above 6 mm and with higher stone density. Given the density of 1100 HU, ESWL may be less effective for this stone.	+	+	+
6	*Further examination* 1. Metabolic evaluation: Since the patient has a history of recurrent stone formation and the composition of past stones is unknown, a metabolic evaluation is crucial. This should include serum calcium, uric acid, phosphate, electrolytes, and a 24-h urine collection for volume, calcium, oxalate, uric acid, citrate, pH, and possibly cystine, depending on initial findings. 2. Coagulation profile: Given the patient’s history of coagulopathy, a detailed coagulation profile including PT, aPTT, INR, and platelet count is essential to assess bleeding risk prior to any surgical intervention. 3. Liver function tests: These tests are necessary due to the patient's hepatomegaly to evaluate liver function and further investigate the cause of hepatomegaly, which could be related to his coagulopathy. 4. Renal function tests: Serum creatinine and eGFR to assess baseline renal function. 5. Imaging: The current CT scan provides good information on stone size and location. Depending on the planned treatment and to assess ureteral anatomy more precisely, additional imaging such as intravenous urography or a renal ultrasound might be beneficial. *Surgical treatment indication* Given the size and number of stones (2.3 cm in the pelvis and up to 1.4 cm in the calyces), surgical treatment is indicated due to the unlikely success of spontaneous passage and the risk of complications such as obstruction, infection, or further impairment of renal function. *Surgical procedure options* 1. PCNL: This is the preferred method for large stones (>2 cm) and staghorn calculi. PCNL involves creating a tract from the skin into the kidney through which instruments can be passed to fragment and remove the stones. Given the size of the largest stone (2.3 cm) and multiple locations within the kidney, PCNL offers the best chance for complete stone removal in a single session. The patient’s coagulopathy must be managed preoperatively to minimize bleeding risks. 2. RIRS: For stones smaller than 2 cm, RIRS could be considered; however, given the size of the largest stone and presence of multiple stones, RIRS may not be the most efficient first choice but could be used adjunctively if residual stones are left after PCNL. 3. ESWL: Typically used for stones <2 cm in size and with a density <1000 HU. Given the size of the patient's stones and their number, ESWL alone is unlikely to be effective but might be considered for smaller residual stones following PCNL.	+	–	+
7	*Treatment options* 1. Observation with MET: Given the size of the stone (10 mm), spontaneous passage is less likely, although MET using alpha-blockers (eg, tamsulosin) can be considered to facilitate stone passage for stones <10 mm. However, the success rate decreases as the stone size approaches 10 mm, especially for stones located in the lower pole. 2. ESWL: (a) ESWL is a noninvasive option that uses shock waves to break the stone into smaller fragments that can be passed more easily. The effectiveness of ESWL can be influenced by the stone's size, density, and location. A stone density of 900 HU is within the range where ESWL could be effective, but lower pole stones have a somewhat lower clearance rate after ESWL due to anatomical challenges in fragment clearance. (b) The distance from the skin to the stone (9 cm) is within an acceptable range for ESWL, and the patient's normal BMI facilitates effective treatment. 3. URS with laser lithotripsy: For stones that are less amenable to ESWL or when ESWL fails, URS with laser lithotripsy is a highly effective treatment option. It involves the use of a small camera (ureteroscope) passed through the urethra and bladder into the ureter or kidney to visualize and directly fragment the stone with a laser. This method is particularly effective for stones in the lower pole and has a high success rate for stones up to 10 mm. 4. PCNL: Although typically reserved for larger stones (>20 mm), PCNL can be considered for lower pole stones >10 mm that are unlikely to be cleared effectively by ESWL or URS. Given this patient's stone size and location, PCNL might be more invasive than necessary, but could be an option if other methods fail or are deemed unsuitable. *Recommended strategy* Given the specific details of this case—a 10-mm lower pole kidney stone with a density of 900 HU in a patient with no contraindications to intervention—the most appropriate initial treatment options would likely be either ESWL or URS with laser lithotripsy. The choice between these two options would depend on factors such as local expertise and availability, patient preference, potential for stone clearance, and consideration of possible complications.	+	–	+
8	*Additional examination* 1. Renal function tests: To assess the overall renal function, including serum creatinine and eGFR. 2. Urinalysis: To check for signs of infection or hematuria. 3. Urine culture: If there is any suspicion of infection, a urine culture should be performed to identify any bacteria present and their antibiotic sensitivities. 4. Metabolic evaluation: Given the size of the stone, a metabolic evaluation including serum calcium, uric acid levels, and a 24-h urine collection for volume, calcium, oxalate, uric acid, sodium, citrate, and pH should be considered to identify any metabolic abnormalities that could have contributed to stone formation. 5. Imaging: A noncontrast CT scan has already been performed. However, in some cases, further imaging such as renal ultrasonography or a contrast-enhanced CT scan (if not contraindicated) might be helpful to evaluate the anatomy of the urinary tract more thoroughly and to assess the presence of any obstruction or other abnormalities. *Surgical tactics* Given the size (19 mm) and density (1300 HU) of the stone, along with the patient's obesity (BMI >30), spontaneous passage is highly unlikely, and active treatment is warranted. The options include the following: 1. PCNL: This is often the first-line treatment for large kidney stones (>15 mm). PCNL involves creating a small tract from the skin into the kidney through which instruments can be passed to fragment and remove the stone. It has a high success rate for large stones but comes with risks such as bleeding and infection. Given the patient's high BMI, there may be technical challenges, but modifications in technique and the use of ultrasound guidance can help mitigate these risks. 2. RIRS: RIRS involves passing a flexible ureteroscope through the bladder and ureter into the kidney to directly visualize and treat the stone with laser lithotripsy. While RIRS is less invasive than PCNL, its efficacy decreases as stone size increases beyond 15 mm. However, it might be considered in certain cases depending on the stone's location and surgeon's expertise. 3. ESWL: ESWL is generally less effective for stones larger than 10 mm and those with high density similar to this one (1300 HU). Additionally, obesity can limit its effectiveness due to difficulty focusing shock waves accurately. Therefore, ESWL is likely not the best option in this case.	+	–	+
9	*Surgical treatment indication* For kidney stones larger than 1 cm, especially those located in the renal pelvis, nonsurgical treatments such as ESWL are often less effective. Given the size of the stone (1.7 cm) and its density (600 HU), which is within the treatable range for ESWL but considering the size and likely effectiveness, more invasive options are typically considered. *Surgical procedure options* 1. PCNL: This is often the preferred method for large stones (>1.5 cm), including those in the renal pelvis. PCNL involves creating a tract from the skin into the kidney through which instruments can be passed to fragment and remove the stone. It offers a high success rate for complete stone removal in a single session for stones of this size. 2. RIRS: For stones smaller than 2 cm, RIRS is also a viable option. It involves using a flexible ureteroscope passed through the bladder and up the ureter into the kidney to directly visualize and treat the stone with a laser. However, for a 1.7-cm stone, RIRS might be less effective than PCNL in terms of achieving complete stone removal in one session. but remains an option depending on the surgeon's assessment and patient's preference. 3. ESWL: Given the size of the stone, ESWL might be considered but is less likely to be effective for stones larger than 1.5 cm in a single session and may require multiple treatments. The stone's density (600 HU) makes it amenable to fragmentation by ESWL, but patient factors and stone size lean toward a more definitive treatment such as PCNL. *Preoperative management* Given the patient's coagulopathy and hepatomegaly, careful preoperative preparation is crucial:1. Coagulopathy correction: Collaborate with a hematologist to optimize the patient's coagulation status before surgery to minimize bleeding risks. 2. Liver function assessment: Ensure that liver function is assessed adequately and managed, given the hepatomegaly, as it could impact coagulation and surgical outcomes. In summary, for a 1.7-cm renal pelvic stone in a patient with coagulopathy and hepatomegaly, PCNL stands out as the most suitable surgical option due to its high success rate for complete stone removal in a single session, with careful preoperative management of coagulopathy being essential.	+	–	+
10	*Risk group for recurrent urolithiasis* Given the patient's clinical presentation and anatomical abnormality (horseshoe kidney), she falls into a higher-risk group for recurrent urolithiasis. Horseshoe kidneys are associated with a higher incidence of stone formation due to altered urinary flow dynamics and possible urinary stasis, which increases the risk of stone formation. Additionally, the observed increased papillary density could suggest Randall’s plaques, which are associated with calcium stone formation *Stone apassednalysis* Yes, it is necessary to submit the stone for mineral composition analysis if it is available. Knowing the stone's composition can guide dietary and medical management to prevent recurrence. For instance, calcium oxalate stones might prompt recommendations to reduce oxalate-rich foods and increase hydration, whereas uric acid stones could lead to dietary modifications to reduce purine intake and possibly the use of medications such as allopurinol. *24-h urine analysis* A 24-h urine collection is essential for this patient to evaluate her risk factors for stone formation and guide further preventive measures. The key parameters to measure include the following: 1. Volume: Aim for >2.5 l/d. Low volume can concentrate stone-forming solutes.2. Calcium: Normal range is 100–300 mg/d for women. Hypercalciuria can contribute to stone formation. 3. Oxalate: Normal range is <45 mg/d. Hyperoxaluria is a risk factor for calcium oxalate stones. 4. Uric acid: Normal range is <600 mg/d for women. Hyperuricosuria can lead to uric acid stones. 5. Citrate: Normal range is >320 mg/d. Hypocitraturia can promote stone formation by reducing stone inhibitor activity. 6. pH: A pH of <5.5 may indicate uric acid stones, while a pH of >7.2 may suggest infection stones (struvite) or cystine stones in the case of cystinuria. 7. Sodium: Recommendations usually aim for <2300 mg/d as high sodium intake can increase calcium excretion. 8. Phosphate: Normal range varies, but hyperphosphaturia can be involved in stone formation. These parameters help identify metabolic abnormalities contributing to stone formation, allowing for targeted dietary and medical interventions.	+	+	+
11	*Risk group for recurrent urolithiasis* This patient would be considered to have a relatively lower risk for recurrent urolithiasis than individuals with a strong family history of stones, multiple episodes of stone passage, or known metabolic disorders. However, having had one stone episode places her at a higher risk than the general population who has never had a stone. The recurrence rate for kidney stones varies, but approximately 50% of individuals may experience another stone within 5–10 yr after their first. *Stone analysis* Yes, it is indeed necessary and highly beneficial to submit the passed stone for a mineral composition analysis if it is available. Knowing the type of stone (eg, calcium oxalate, uric acid, struvite, or cystine) can significantly influence both dietary and medical management aimed at preventing future stones. For instance, management strategies differ markedly between calcium oxalate and uric acid stones in terms of dietary recommendations and potential pharmacotherapy. *24-h urine analysis* A 24-h urine collection is recommended for this patient to identify any metabolic abnormalities that could increase her risk of forming more stones. The key parameters to measure and their reference values include the following: 1. Volume: Aim for >2.5 l/d to reduce the concentration of lithogenic substances. 2. Calcium: normal range is 100–300 mg/d for women. Hypercalciuria can contribute to stone formation. 3. Oxalate: A level of <45 mg/d is considered normal. Elevated levels can indicate a risk for calcium oxalate stones. 4. Uric acid: Levels of <800 mg/d for men and <750 mg/d for women are normal. High levels can lead to uric acid stones. 5. Citrate: A level of >320 mg/d is desirable. Low levels can promote stone formation as citrate is an inhibitor of stone formation. 6. pH: The normal urinary pH ranges from 4.5 to 8.0. A persistently low or high pH can indicate a risk for certain types of stones (eg, uric acid stones at low pH and struvite stones at high pH). 7. Sodium: While not always measured, excessive sodium intake can increase calcium excretion and stone risk. Recommendations usually aim for <2300 mg/d. 8. Phosphate, magnesium, and others: Depending on initial findings, additional tests might be warranted to further clarify the risk profile.	+	+	+

BUN = blood urea nitrogen; BMI = body mass index; CT = computed tomography; eGFR = estimated glomerular filtration rate; ESWL = extracorporeal shockwave lithotripsy; IV = intravenous; MET = medical expulsive therapy; NSAID = nonsteroidal anti-inflammatory drug; PCN = percutaneous nephrostomy; PCNL = percutaneous nephrolithotomy; RIRS = retrograde intrarenal surgery; URS = ureteroscopy; UTI = urinary tract infection.

Although all responses were provided with information that corresponded to EAU guidelines, six of the 11 answers were associated with missed guideline–provided parts, and incorrect data were given in eight of the 11 answers.

### 3.1 Question 1: Relates to imaging modality in flank pain

Even with such a brief question, a detailed answer was provided for each item. Thus, it is correctly determined that the first-line imaging modality for suspected urolithiasis is a noncontrast helical (spiral) computed tomography (CT) scan of the abdomen and pelvis. In addition, although not stated in the question, the chat notes the importance of using other research methods (in particular, ultrasound scans) in certain cohorts of patients, such as pregnant women and children. The description of the laboratory test results was consistent with the guidelines. However, phosphorus level is not exactly indicated as a recommended parameter in laboratory examinations.

### 3.2 Question 2: Relates to investigations of incidentally found renal stone

GPT-4 describes the laboratory minimum for a patient with an asymptomatic stone in the pelvis of the left kidney. Not only does the chat response directly indicate the necessary parameters, it also provides brief descriptions to support it. The chat’s reasoning at the end of the answer confirms the good academic nature of GPT-4. However, omissions remain. Therefore, in the “24-h urine collection” section, there is no mention of magnesium.

### 3.3 Question 3: Relates to urgent treatment in a patient with recurrent urinary tract infection and 6 cm stone in solitary kidney

GPT-4 describes the available approaches, which are better to choose in this case. However, there is only a list of necessary actions without a correct sequence. Moreover, specific antibiotics and their dosages were not specified. Although surgical tactics have been described, these may not always be appropriate.

### 3.4 Question 4: Relates to management of vesicoureteric junction stone

The GPT-4 response appears to be erudite for several reasons. First, hydration is also recommended to stabilize the patient’s condition; however, given renal colic in this clinical scenario, the chat warns that “aggressive hydration is not recommended as it may increase renal pelvis pressure and pain without improving stone passage rates.” Second, the importance of performing a CT scan for the diagnosis of urolithiasis is discussed. Third, the localization of the stone and its size were correctly analyzed from the perspective of the need for drainage of the upper urinary tract. The chat’s reasoning regarding the composition of the stone in the anamnesis also looks impressive, which confirms the use of up-to-date information when training this model. Finally, follow-up was described very closely to the guidelines. However, all drug therapies are indicated in the abstract, without specifying the drugs.

### 3.5 Question 5: Relates to management of renal colic related to proximal ureteric stone

In general, the chat response regarding the treatment strategy in this case was adequate and mostly corresponded with the guidelines. The use of nonsteroidal anti-inflammatory drugs or opioids for pain relief is recommended even in the absence of specific drugs. There is no advice on how to use antibiotics, which corresponds to the clinical picture and does not mislead the user. Analogous to the previous question, hydration was also mentioned. However, in this case, the chat did not warn about the risk of increased stretching of the kidney cavity and the development of complications. Moreover, the chat initially recommends medical expulsion therapy (MET) with tamsulosin but later asserts that spontaneous stone passage is less likely, a statement that is indeed accurate. Treatment in the form of ureteroscopy (URS) is correct and recommended, and is the reason for the lower effectiveness of extracorporeal shockwave lithotripsy (ESWL). However, its effectiveness decreases with stone sizes above 6 mm, which is confusing and not mentioned in the guidelines directly.

### 3.6 Question 6: Relates to management of multiple complex renal stones with coagulopathy

The chat correctly recommended further patient evaluation. This is not entirely in accordance with the recommendations, but with the potential need for other instrumental diagnostic methods to clarify the anatomy of the upper urinary tract, which is reasoned in connection with the patient’s hepatomegaly. The list of possible surgical tactics outlines their advantages and disadvantages accurately, considering the size and location of the stones in a specific patient. However, the very first recommendation is “24-h urine collection for volume, calcium, oxalate, uric acid, citrate, pH, and possibly cystine, depending on initial findings.” This is incorrect, and it is not clear which initial findings are meant.

### 3.7 Question 7: Relates to management of 1 cm lower pole stone

Despite the informativeness of the answer, for some reason, the chat begins to mention MET as a potential treatment for stones in the lower calyceal group, although it further says that its effectiveness is low. However, the answer devoted to the choice of specific surgical tactics follows the recommendations and considers all the aspects mentioned in the question. The chat correctly notes that not only the size of the stone, but also the skin-to-stone distance is favorable for performing ESWL, which confirms the fact that modern and relevant information was used when training it. Finally, the chat discusses the possibility of performing ESWL and URS in this case, and explains what the final choice may be related to, which also corresponds to the recommendations.

### 3.8 Question 8: Relates to 19 mm lower pole stone in an obese patient detected incidentally

The chat, although without specifics, correctly discusses the laboratory and instrumental diagnostics of a patient before the operation. Moreover, the possibility of performing contrast-enhanced CT to detail the urinary tract anatomy in specific cases was discussed. A low probability of spontaneous passage of the stone, considering its size, was correctly noted. The following are the descriptions of possible surgical tactics. However, for some reason, the chat is discussing the effectiveness of percutaneous nephrolithotomy (PCNL) and retrograde intrarenal surgery (RIRS) using a cutoff of 15 mm; however, this value is not mentioned in the guidelines, and a different gradation is used.

### 3.9 Question 9: Relates to 1.7 cm renal pelvic stone with coagulopathy

In this case, the chat immediately noted the need for invasive surgery due to the size of the stone and the ineffectiveness of ESWL. Among PCNL and RIRS, the chat correctly notes the greater prospects for performing the former; however, as in the previous question, it uses 15 mm as a boundary value for choosing surgical tactics, whereas when discussing RIRS, the boundary value is 20 mm, which exactly corresponds to the gradation in guidelines. It should also be noted that the chat did not ignore the presence of coagulopathy and hepatomegaly, and emphasized the importance of stabilizing the patient’s condition before any intervention.

### 3.10 Question 10: Relates to metaphylaxis in a high-risk patient who spontaneously passed a stone

In the question regarding metaphylaxis in a patient with a horseshoe kidney, GPT-4 correctly classified the patient into a high-risk group for stone formation and recurrence. Moreover, the chat correctly noted the importance of determining the mineral composition of the stone to choose the most optimal tactics to prevent recurrence. However, the main questions remain regarding references to 24-h urinalysis. Moreover, not all the listed parameters coincide with those specified in the guidelines. Finally, the problem of inconsistency between measurement units and guidelines remains unresolved. This indicates that the EAU guidelines for urolithiasis are less likely to be used for GPT-4 education.

### 3.11 Question 11: Relates to metaphylaxis in a low-risk patient who spontaneously passed a stone

GPT-4 correctly noted that the patient in this case had a low risk of early stone formation recurrence. However, when studying the chat’s competence in interpreting 24-h urine parameters, more questions arise when compared with the answer to the previous question. In this case, the threshold value of uric acid for women was 750 mg/d (instead of the 600 given in question no. 10). All other nuances of the GPT-4 answers were identical to those given in the previous scenario.

## 4 Discussion

To the best of our knowledge, this is the first study to directly assess the correctness of answers in GPT-4 related to the diagnostic, treatment, and follow-up urolithiasis workflow from the point of view of a urologist. As noted earlier, we focused on the initial response for each request without additional clarification, simulating the process of using these chat models by an inexperienced urologist, for whom such generative AI-based models are especially important as tools for supporting medical decision-making. GPT-4 is associated with a model size of 170 trillion parameters and provides support for the picture inputs. However, it is clear that EAU guidelines are not used directly in the training process of these versions. It is imperative for developers of generative AI models such as GPT-4 to use appropriate and specialized guidelines, as it is evident that these tools will readily be accessible to both patients and clinicians. Disregarding this truth can justifiably be regarded as a bias. An AI bias remains a risk, as the model’s responses may reflect biases in its training data, potentially leading to inequitable health care outcomes [8]. Continuous monitoring and mitigation of these biases are essential to uphold fairness and justice in medical care. Considering the results of the analysis of the responses received, we can formulate advice on the correct use of GPT-4 and the further development of this technology:

1. When discussing urolithiasis-associated clinical scenarios, sufficient knowledge of the current guidelines is required to understand the errors in the GPT-4 response.
2. It is important to treat GPT-4 as a young assistant and not as a mentor, whose opinion is decisive in the decision-making process.
3. It is relatively safe to use GPT-4 knowledge in the initial diagnostic flow of patients suspected of having stones within the urinary tract and during treatment planning; however, its understanding of all the nuances of metaphylaxis leaves much to be desired and is far from the dogma given in the EAU guidelines.
4. It is necessary to know all the medications used in the treatment of patients with urolithiasis since the GPT-4 answers do not contain specific recommendations.
5. It is necessary to know all the parameters of laboratory tests (both blood and urine tests) to fully assess the health status of patients with urolithiasis. In this case, GPT-4 is suitable only for discussing individual groups of analyses.
6. Surgical strategy and algorithm were not always in alignment with the EAU guidelines.

Future research should focus on enhancing GPT-4's alignment with clinical guidelines and improving its interpretative accuracy. Developing specialized training datasets that incorporate comprehensive and updated clinical guidelines, such as those from the EAU, will be crucial. Collaborations between AI developers and clinical experts can further refine the model’s responses. Longitudinal assessments of GPT-4’s performance in real-world clinical settings, evaluating its impact on patient outcomes and workflow efficiency, are necessary. Comparative research with other AI tools and traditional decision-making processes will provide deeper insights into the benefits and limitations of AI in urology. Continuously updating the AI with the latest medical knowledge and clinical feedback will enhance its effectiveness in health care. For example, Manolitsis et al [9] assessed the efficacy of a ChatGPT API 3.5 Turbo model compared with a standard model in supporting urologists in obtaining precise and reliable medical information. The API was accessed using a Python script written particularly for this study and based on 2023 EAU guidelines in PDF format. This custom-trained model provides clinicians with more precise and rapid responses concerning specific urologic issues, thereby assisting them in providing better patient care than the existing standard model.

Furthermore, any guidance for the use of generative AI models, including GPT-4, should be accompanied by a cautionary note on the ethical and legal implications of using these as an assisting tool in clinical practice [10]. The medical community is witnessing the rapid development in AI, which appears to be very promising and beneficial to both doctors and patients [11]. However, there is another side to the coin that conceals a significant number of difficulties and hazards that must be considered and resolved prior to any active integration of AI into daily practice. The best explanation of this is the “Collingridge dilemma,” where innovation is accompanied by a progressive revelation of unanticipated societal consequences that quickly outpace our ability to manage and ameliorate them [12]. Therefore, it is important to highlight the importance of the ethical and legal subjects every time we combine GPT-4 with our medical decisions. Working with GPT-4 requires specifying personal data about the patient’s health in the dialogue. Ethically, concerns about patient privacy and data security are paramount in this case, given the model's need to process patient data to generate the response. Ensuring that these data are anonymized and protected against breaches is crucial [13]. Furthermore, the typical patient consent form makes no mention of the use of personalized patient data in the creation or use of AI models. Patients are entitled to thorough information about their diagnoses, treatment processes, potential risks, and data privacy concerns. Individuals have the freedom to deny treatment and must be informed of the accountability system in the event of an AI system malfunction [14]. The patient has the right to know how GPT-4 bases its advice on a specific request, which highlights the relevance of the model's transparency. A fundamental problem connected to the transparency of AI derives from the complexity of AI algorithms, particularly those produced from deep learning models, which are commonly referred to as “black boxes” [15]. The lack of transparency afforded by the neural network prevents not only patients, but also urologists from gaining a full understanding of its internal mechanisms. As demonstrated in our research, knowledge of GPT-4 is sufficient at the outset of assessing patients with urolithiasis and as a junior helper. If the responses from GPT-4 are used as a basis, and this results in a negative outcome, the issue of accountability and liability is critical [16]. If a patient is harmed due to an AI-generated recommendation, it is unclear whether the responsibility lies with the health care provider, the institution, or the AI developers. This legal ambiguity necessitates clear regulatory frameworks. Recent initiatives, such as the European Union’s proposed AI Act, aim to establish standards for AI deployment in high-risk sectors such as health care, emphasizing transparency and explainability [17]. Implementing such frameworks globally would help ensure that AI tools such as GPT-4 are used safely and responsibly, balancing innovation with the protection of patient rights and trust in the health care system.

This study has some limitations. Only the initial responses of GPT-4 were analyzed without clarifying the questions, which could theoretically hinder the understanding of the true competence of the chat. Only the GPT-4 model was analyzed, whereas there were a sufficient number of other representatives of the group of generative AI-based models. In addition, there are other recommendations with which compliance could be greater or less. Nevertheless, this work is important for understanding the vulnerabilities of the latest version of GPT-4 when used by a doctor in real practice.

## 5 Conclusions

While GPT-4 demonstrates notable capability to address patient inquiries regarding urolithiasis, its utility in answering more specific, nuanced questions from urologists remains limited. The model shows proficiency in initial diagnostics and treatment planning, aligning reasonably well with the EAU guidelines in these areas. However, significant gaps exist in its understanding of surgical planning, metaphylaxis, and precise application of guidelines. These findings highlight the necessity for careful interpretation by medical professionals and indicate that GPT-4 should be seen as an adjunct tool rather than a primary decision-maker. Future improvements should focus on incorporating comprehensive and up-to-date clinical guidelines directly into the training data, fostering closer collaborations between AI developers and clinical experts, and refining the model continuously based on real-world clinical feedback. Additionally, addressing the ethical and legal challenges associated with AI in health care is crucial to ensure its safe and responsible use. Such efforts are essential to enhance the accuracy, reliability, and clinical relevance of AI tools in urology.

  ***Author contributions*:** Patrick Juliebø-Jones had full access to all the data in the study and takes responsibility for the integrity of the data and the accuracy of the data analysis.

  *Study concept and design*: Talyshinskii, Somani, Juliebø-Jones, Zhanbyrbekuly, Tzelves, Naik, Adhikari, Hameed.

*Acquisition of data*: Talyshinskii, Juliebø-Jones.

*Analysis and interpretation of data*: Talyshinskii, Juliebø-Jones, Zhanbyrbekuly, Naik, Adhikari, Hameed, Somani.

*Drafting of the manuscript*: Talyshinskii, Somani.

*Critical revision of the manuscript for important intellectual content*: Juliebø-Jones, Tzelves, Zhanbyrbekuly, Naik, Hameed, Adhikari, Somani.

*Statistical analysis*: Talyshinskii.

*Obtaining funding*: None.

*Administrative, technical, or material support*: None.

*Supervision*: Somani, Juliebø-Jones.

*Other*: None.

  ***Financial disclosures:*** Patrick Juliebø-Jones certifies that all conflicts of interest, including specific financial interests and relationships and affiliations relevant to the subject matter or materials discussed in the manuscript (eg, employment/affiliation, grants or funding, consultancies, honoraria, stock ownership or options, expert testimony, royalties, or patents filed, received, or pending), are the following: None.

  ***Funding/Support and role of the sponsor*:** None.
